# Puzzling Papillomas: A Case of an Intraductal Papilloma Mimicking an Abscess

**DOI:** 10.7759/cureus.52393

**Published:** 2024-01-16

**Authors:** Siya Patil, Christine Chen

**Affiliations:** 1 Department of Diagnostic, Molecular and Interventional Radiology, Mount Sinai Medical Center, New York, USA

**Keywords:** benign breast condition, breast cancer research, radiology & imaging, intraductal papilloma, breast disease

## Abstract

Intraductal papillomas (IDPs) are benign tumors found within breast ducts. Clinicians should be familiar with IDPs given their association with atypical and neoplastic lesions. In our case, the patient was initially diagnosed with and treated for an abscess given clinical symptoms of breast pain, erythema, and swelling, but upon returning to the clinic a year later due to persistent symptoms, she was found to have an IDP. This case underscores the importance of atypical imaging features and close follow-up when evaluating breast lesions.

## Introduction

Intraductal breast papillomas are benign tumors that fall within the spectrum of papillary breast lesions [1]. Despite constituting less than 2% of breast lesions [2], they are important as they are high-risk malignancy precursor lesions [3]. Most patients with papillomas are asymptomatic but they can occasionally present with a palpable abnormality and bloody or clear nipple discharge [1]. Timely diagnosis is essential, as upgrade rates to malignant and pre-malignant lesions range from 1.6-20% [3-6]. Our case presents a challenging dilemma of an intraductal papilloma (IDP) initially misdiagnosed as an abscess.

## Case presentation

**Figure 1:**
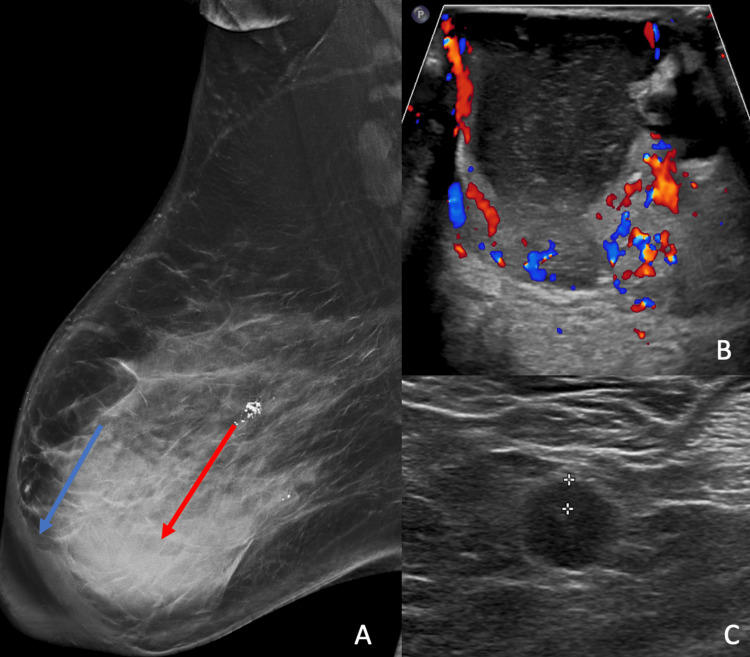
A: Diagnostic mammogram shows a retroareolar right breast mass (red arrow) and associated skin and trabecular thickening (blue arrow); B: Diagnostic ultrasound revealed a hypervascular, complex cystic mass; C: Prominent lymph nodes with concentric cortical thickening, measuring up to 0.4 cm, in the ipsilateral axilla were also found

A 57-year-old postmenopausal female presented to the breast surgery clinic with right breast swelling, pain, and bloody nipple discharge for three months. Physical exam revealed diffuse right breast thickening and erythema, but no palpable masses. She was subsequently referred for diagnostic imaging. A diagnostic mammogram revealed a 7.5 cm retroareolar mass with associated skin and trabecular thickening (Figure 1A). The left breast did not reveal any suspicious findings (not shown). On diagnostic ultrasound, there was a hypervascular complex cystic mass with thick, irregular walls measuring 7.5 x 6.8 x 7.1 cm (Figure 1B). A few prominent lymph nodes with concentric cortical thickening were identified in the right axilla (Figure 1C). These findings were suspicious, so the lesion was categorized as BIRADS 4 (Breast Imaging-Reporting and Data System), and an ultrasound-guided core biopsy was recommended.

The patient opted for a skin punch biopsy of the right breast in the surgeon’s office, which yielded inflammatory changes and neutrophils. Given the high clinical suspicion of breast abscess, incision and drainage of the right breast mass was performed, which yielded purulent fluid. The patient was prescribed oral antibiotics with clinical improvement and instructed to follow up in the clinic in three months.

**Figure 2:**
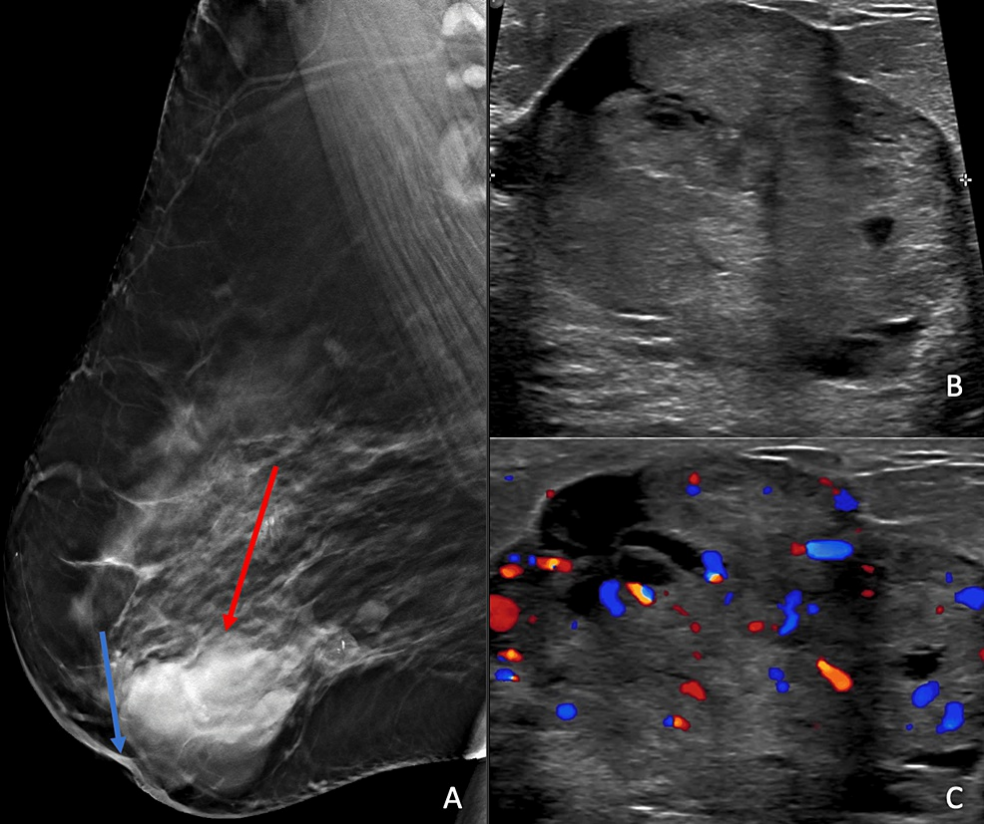
A: Right breast mammogram a year later shows skin thickening, new nipple retraction (blue arrow), and a mass (red arrow); B and C: Ultrasound of the right breast revealed a corresponding hypervascular, hypoechoic mass

The patient was lost to follow-up and returned a year later with a new palpable mass in the right breast, new nipple retraction, as well as persistent right-sided bloody nipple discharge. A diagnostic mammogram was performed and revealed a mass in the right central inner breast associated with new nipple retraction (Figure 2A). On ultrasound, there was a corresponding 4.0 x 3.5 x 1.3 cm irregular hypoechoic mass with internal vascularity (Figure 2B and Figure 2C). 

These findings were deemed suspicious and an ultrasound-guided core biopsy was performed, which yielded a benign intraductal papilloma. Given the large size and nipple retraction, as well as the potential risk of under-sampling, surgical excision was advised.

**Figure 3:**
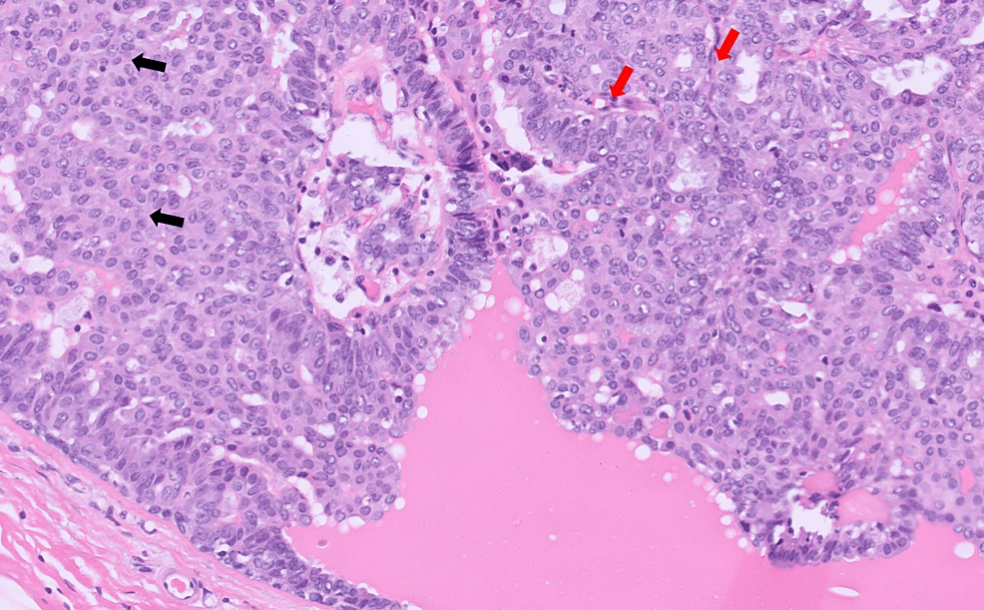
This slide shows the intraductal papilloma with usual ductal hyperplasia (black arrows) and fibrovascular cores (red arrows)

Wire localized excision of the right breast mass was performed with final surgical pathology revealing a benign intraductal papilloma (Figure 3). The patient will be seen annually in the high-risk screening clinic.

## Discussion

Our case demonstrates an unusual imaging appearance and clinical presentation of a benign IDP with abscess formation. Intraductal papillomas are benign, high-risk lesions, which can be categorized as solitary versus multiple intraductal papillomas. Solitary IDPs, as in our case, are located in the retroareolar region and may present with clear or bloody nipple discharge [1,5]. Multiple intraductal papillomas are less common, tend to arise from the terminal ductal lobular unit, and may present as a palpable abnormality [7]. Furthermore, there is an increased risk of coexisting atypical and neoplastic lesions with multiple IDPs, compared with solitary IDPs [7].

On mammography, IDPs may be imaging-occult given dense breast tissue in the retroareolar region [7]. When mammographically evident, IDPs usually present as round or oval masses with circumscribed margins, or less commonly as grouped benign-appearing calcifications [4,5,7]. On ultrasound, IDPs appear as an intraductal mass and may have an associated feeding vessel [7]. On MRI, smaller lesions may be occult while larger IDPs can appear as enhancing masses with variable intraductal components [5,7]. The lesions may enhance irregularly or uniformly and can have washout or plateau kinetics [7]. Finally, on galactography, IDPs appear as mural-based filling defects with smooth or lobulated margins [7]. In terms of management, IDPs are generally surgically excised because they are high-risk precursor lesions [1]. Upgrade rates to malignancy range from 1.6% to 20% [3-6] depending on the presence of atypia. Alternatively, if surgery is not desired, radiologic follow-up may be an appropriate method of surveillance for select cases of IDPs that are small (less than 1.0 cm) and without atypia [8].

To our knowledge, no other examples of an IDP with inflammatory characteristics exist in the literature. The case was challenging as the patient initially presented with symptoms of infection and histopathology from the initial skin punch biopsy confirmed infectious etiology. Furthermore, incision and drainage revealed yellow purulent fluid and the patient clinically improved after a course of oral antibiotics. It is possible that the large size of the mass led to duct obstruction and superimposed infection of the IDP. Unfortunately, the patient was lost to follow-up for one year and the diagnosis of IDP was made via core biopsy more than one year after initial presentation. This case demonstrates the importance of close clinical follow-up for patients suspected of breast abscess while illustrating a highly unusual imaging appearance of an IDP as a retroareolar mass associated with skin thickening and nipple retraction.

## Conclusions

IDPs are benign entities with a wide spectrum of imaging appearances. Biopsy should be performed for most cases given the variable imaging appearance and characterization as high-risk precursor lesions. Finally, if a patient is diagnosed with a breast abscess, follow-up imaging to resolution can be beneficial.
